# Generation of Skeletal Muscle Organoids from Human Pluripotent Stem Cells

**DOI:** 10.21769/BioProtoc.4984

**Published:** 2024-05-05

**Authors:** Urs Kindler, Holm Zaehres, Lampros Mavrommatis

**Affiliations:** 1 Ruhr University Bochum, Medical Faculty, Institute of Anatomy, Department of Anatomy and Molecular Embryology, Bochum, Germany; 2 Ruhr University Bochum, Medical Faculty, Department of Neurology with Heimer Institute for Muscle Research, University Hospital Bergmannsheil, Bochum, Germany

**Keywords:** Skeletal muscle, Organoids, Myogenesis, iPS cells, Satellite cells, PAX7, Disease modeling, Muscle dystrophy

## Abstract

Various protocols have been proven effective in the directed differentiation of mouse and human pluripotent stem cells into skeletal muscles and used to study myogenesis. Current 2D myogenic differentiation protocols can mimic muscle development and its alteration under pathological conditions such as muscular dystrophies. 3D skeletal muscle differentiation approaches can, in addition, model the interaction between the various cell types within the developing organoid. Our protocol ensures the differentiation of human embryonic/induced pluripotent stem cells (hESC/hiPSC) into skeletal muscle organoids (SMO) via cells with paraxial mesoderm and neuromesodermal progenitors’ identity and further production of organized structures of the neural plate margin and the dermomyotome. Continuous culturing omits neural lineage differentiation and promotes fetal myogenesis, including the maturation of fibroadipogenic progenitors and PAX7-positive myogenic progenitors. The PAX7 progenitors resemble the late fetal stages of human development and, based on single-cell transcriptomic profiling, cluster close to adult satellite cells of primary muscles. To overcome the limited availability of muscle biopsies from patients with muscular dystrophy during disease progression, we propose to use the SMO system, which delivers a stable population of skeletal muscle progenitors from patient-specific iPSCs to investigate human myogenesis in healthy and diseased conditions.

Key features

• Development of skeletal muscle organoid differentiation from human pluripotent stem cells, which recapitulates myogenesis.

• Analysis of early embryonic and fetal myogenesis.

• Provision of skeletal muscle progenitors for in vitro and in vivo analysis for up to 14 weeks of organoid culture.

• In vitro myogenesis from patient-specific iPSCs allows to overcome the bottleneck of muscle biopsies of patients with pathological conditions.

**Graphical overview**


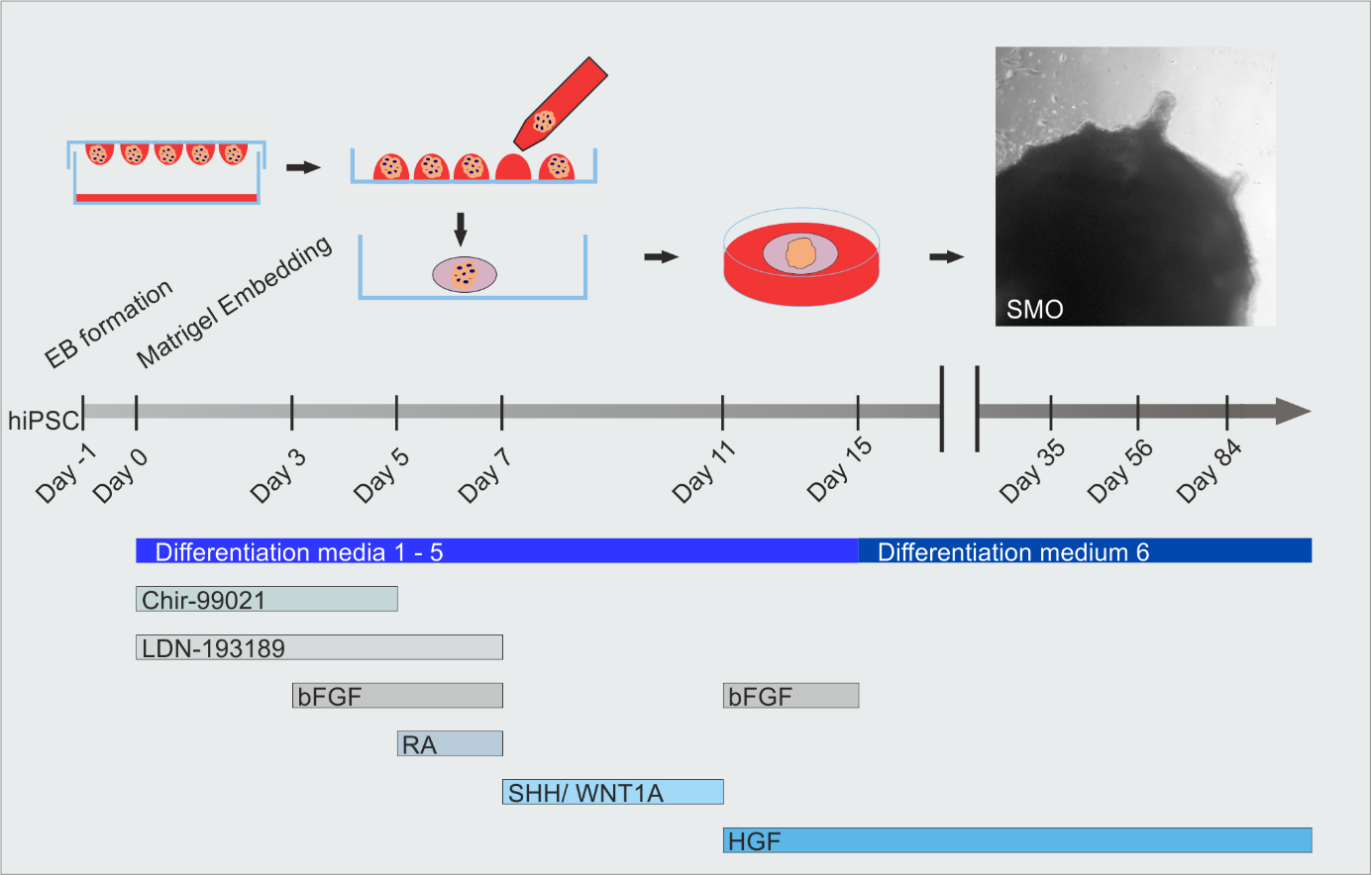


**Skeletal muscle organoid induction and timeline of differentiation media applications and growth factor compositions**

## Background

Human stem cell–based in vitro models are becoming more frequently used specifically for human disease modeling, increasingly substituting artificial animal models. Using patient-derived cells for reprogramming opens the opportunity to generate cell lines from each genetic disease to study the specific phenotype and their underlying molecular mechanisms [1]. Even the U.S. Food and Drug Administration Modernization Act 2.0 in 2022, is paving the way for primarily using cell cultures for testing novel drugs.

For several years, various protocols have been proven effective in the directed differentiation of pluripotent stem cells (PSCs) into skeletal muscles and used to study the developmental stages of muscle differentiation [2–7], reviewed in Kim and Perlingeiro, Chien et al. and Yan et al. [8–10].

Importantly, the PSCs need to be guided toward paraxial mesoderm, which can be achieved by WNT pathway activation and BMP pathway inhibition along with FGF [2,3,11,12]. The early paraxial mesoderm is specified by WNT activation, while BMP inhibition prevents its shift toward the lateral-plate mesoderm. The transition from the posterior to the anterior somitic mesoderm can be confirmed by PAX3 expression [3]. As stated by the protocol of Chal and colleagues, the treatment by trophic factors such as bFGF, IGF, and HGF allows the PAX3 population to induce the myogenic program [4].

Recently, 3D differentiation has come into focus to mimic the environmental heterogeneity and the interactions among the different cell types and within tissues. In general, the maintenance and regeneration of tissue is aided by creating stem cell niches, which promote stem cell regeneration through their microenvironments. In case of skeletal muscles, various cell types are necessary to establish the stem cell niche–like fibroadipogenic progenitors, endothelial cells, pericytes, and macrophages, as well as transient neural crest structures that influence the skeletal muscle stem cell fate through various extracellular signals [13,14]. Besides establishing the stem cell niche, organoids can resemble early embryonic development and fetal cell maturation. These processes are essential for the initial pathological signs and early phenotypes in disorders such as Duchenne muscular dystrophy (DMD) [15,16].

The well-documented progression of diseases narrows the focus of the investigations to specific tissue and cell types. The prolongation of cultivation in vitro represents a challenge that overcomes embryonic gene expression and allows the observation of myogenesis until the onset of the disease in fetal or adult age. However, this appears mandatory to model genetic disorders like muscular dystrophies that have a relatively late onset. To overcome the lack of continued observation along myogenesis and disease onset, long-term culture approaches are essential to examine the mechanisms leading to muscle loss during myogenesis. We have established a 3D skeletal muscle organoid (SMO) system, which delivers a stable population of skeletal muscle progenitors to investigate human myogenesis during early embryonic and fetal development [17,18]. Induction of paraxial mesoderm is mediated by BMP inhibition, Wnt activation, and bFGF application as in other 2D protocols [4]. The application of retinoic acid, Shh, and WNT1A distinguishes this protocol from other 2D protocols and specifically leads to anterior somitic mesoderm and neural crest formation. The early Matrigel embedding is the decisive difference to all 2D protocols. In comparison to 2D protocols, our 3D protocol patterns Pax7-positive skeletal muscle progenitor cells with dormant, activated, and committed signatures of late fetal stages partially overlapping with adult satellite cell developmental scoring, which can be maintained for up to 14 weeks of culturing. Our 3D differentiation protocol does not go beyond 2D protocols to provide maturated physiologically responsive skeletal muscle cells, which we have demonstrated with the electrophysiological recording of organoid-derived cells of different origins [18].

However, structural distinctions like the posterior paraxial mesoderm on day 5, specified neural crest dermomyotome on day 17, myogenic progenitor migration on day 23, and neural crest lineage arrest on day 35 cannot be similarly mimicked with PSC-differentiation in 2D protocols. Recently, three other groups have described protocols for skeletal muscle development within 3D organoid differentiation systems [19–21]. In comparison to these protocols, we have focused on the myogenic progenitor cell identity in comparison to satellite cells by scRNAseq in greater detail (e.g., dormant, activated, and committed signatures) [18]. We see the strength of our system as being able to retain Pax7-positive myogenic progenitors/satellite-like cells constantly even during long-term cultivation to study their alterations in muscular dystrophies when generated from patient-derived induced pluripotent stem cells (iPSCs).

## Materials and reagents

**Biological materials**

1. Human CD34 cord blood iPSCs [22]
2. Human LGMD2A and LGMD2A-isogenic iPSCs [23]
3. Human DMD iPSCs [24,25]
4. Human CD34 cord blood iPSC [26] (Gibco^TM^ Episomal iPSC Line, catalog number: A18945)
5. HsdCpb: NMRI-Foxn1nu mice (NMRI-Foxn1nu/Foxn1nu, Janvier, St Berthevin Cedex, France)

**Reagents**

1. DMEM/F12 (Gibco^TM^, catalog number: 21331020)
2. ITS-G (Gibco^TM^, catalog number: 41400045)
3. ITS-X (Gibco^TM^, catalog number: 51500056)
4. L-Glutamine (Gibco^TM^, catalog number: 25030081)
5. Nonessential amino acids (NEAA) (Gibco^TM^, catalog number: 11140050)
6. Retinoic acid (Sigma-Aldrich, catalog number: R2625)
7. LDN-193189 (Sigma-Aldrich, catalog number: 1062368-24-4)
8. CHIR-99021 (Biogems, catalog number: 2520691)
9. Recombinant human FGF-basic (bFGF) (Peprotech, catalog number: 100-18B)
10. Recombinant human HGF (Peprotech, catalog number: 100-39H)
11. Recombinant human Sonic Hedgehog (SHH) (Peprotech, catalog number: 100-45)
12. Recombinant human Wnt-1 (WNT1A) (Peprotech, catalog number: 120-17)
13. Penicillin/streptomycin (P/S) (Gibco^TM^, catalog number: 15070063)
14. Poly-(methacrylacid-2-hydroxyethylester) (Sigma-Aldrich, catalog number: P3932)
15. Polyvinyl alcohol (PVA) (Sigma-Aldrich, catalog number: 363065)
16. TeSR^TM^-E8^TM^ (StemCell Technologies, catalog number: 05990)
17. StemFlex^TM^ medium (Thermo Fischer Scientific, catalog number: A3349401)
18. TrypLE^TM^ Select (Gibco^TM^, catalog number: 12563011)
19. Y-27632 (StemCell Technologies, catalog number: 72304)
20. Papain from papaya latex (Sigma-Aldrich, catalog number: P4762)
21. Corning^®^ Matrigel^®^ growth factor reduced (Corning, catalog number: 354230)
22. Anti-Brachyury (R&D Systems, 1:250, catalog number: AF2085)
23. Anti-TBX6, 1:200 (Abcam, catalog number: ab38883)
24. Anti-PAX3, 1:250 (DHSB, catalog number: N/A)
25. Anti-PAX7, 1:250 (DHSB, catalog number: N/A)
26. Anti-SOX10, 1:125 (R&D Systems, catalog number: AF2864)
27. Anti-TFAP2A, 1:100 (DHSB, catalog number: 3B5)
28. Anti-FastMyHC, clone MY-32, 1:300 (Sigma-Aldrich, catalog number: M4276)
29. Anti-SOX2, clone Btjce, 1:100 (Thermo Fisher Scientific, catalog number: 14-9811-82)
30. Anti-Dystrophin, 1:20 (Leica, catalog number: NCLDYS1)
31. Anti-Lamin A + Lamin C antibody, 1:150 (Abcam, catalog number: ab108595)
32. PE anti-human CD82 antibody (BioLegend, catalog number: 342103)
33. Goat anti-Mouse IgG (H + L) cross-adsorbed secondary antibody, Alexa Fluor 568, 1:1,000 (Invitrogen, catalog number: A-11063)
34. Goat anti-Rabbit IgG (H + L) highly cross-adsorbed secondary antibody, Alexa Fluor Plus 488, 1:1,000 (Invitrogen, catalog number: A32731)
35. HISTOPRIME normal goat serum, sterile (NGS) (BIOZOL, catalog number: LIN-ENG1000-100)
36. Rhodamine Red^TM^-X (RRX) AffiniPure Goat Anti-Mouse IgG, Fcγ subclass 1 specific, 1:100 (Jackson ImmunoResearch Laboratories, catalog number: 115-295-003)
37. Alexa Fluor 488, Goat Anti-Rat IgG (H + L) cross-adsorbed secondary antibody, 1:500 (Thermo Fisher Scientific, catalog number: A-11006)
38. Alexa Fluor 488, Donkey Anti-Mouse IgG (H + L) cross-adsorbed secondary antibody, 1:500 (Thermo Fisher Scientific, catalog number: A32766)
39. Alexa Fluor 488, Donkey Anti-Goat IgG (H + L) cross-adsorbed secondary antibody, 1:500 (Thermo Fisher Scientific, catalog number: A32814)
40. Alexa Fluor 568, Donkey Anti-Rabbit IgG (H + L) cross-adsorbed secondary antibody, 1:500 (Thermo Fisher Scientific, catalog number: A10042).
41. Cardiotoxin (CTX) (Sigma-Aldrich, catalog number: 217503-1MG)
42. Sodium citrate, dihydrate (Fisher Scientific, catalog number: 15538154)
43. Ethylenediamine tetraacetic acid disodium salt dihydrate (CARL ROTH, catalog number: 8043.2)
44. Citric acid monohydrate (Sigma-Aldrich, catalog number: C7129)
45. Single-cell 3’ Library & Gel Bead kit v3 (10× Genomics, catalog number: PN-1000075)
46. Single-cell B Chip kit (10× Genomics, catalog number: PN-1000073)
47. i7 Multiplex kit (10× Genomics, catalog number: PN-120262)
48. Bovine albumin (BSA) fraction V (7.5% solution) (Gibco^TM^, catalog number: 15260037)
49. Glycine (Sigma-Aldrich, catalog number: G8898)
50. L-cysteine (Sigma-Aldrich, catalog number: 168149)
51. Paraformaldehyde (PFA) (Sigma-Aldrich, catalog number: 158127)
52. Sucrose (Sigma-Aldrich, catalog number: 84100)
53. Tween-20 (Sigma-Aldrich, catalog number: 11332465001)
54. Tissue-Tek^®^ O.C.T. compound (Sakura Finetek, catalog number: SA62550-01)
55. Triton X-100 (Sigma-Aldrich, catalog number: X100-100ML)
56. DAPI ready-made solution (Sigma-Aldrich, catalog number: MBD0015-5ML)
57. PBS (10×), pH 7,4 (Gibco^TM^, catalog number: 70011044)

**Solutions**

1. Coating solution (100 mL) (see Recipes)
2. Differentiation media 1 (Di-CL) (100 mL) (see Recipes)
3. Differentiation media 2 (Di-CLF) (100 mL) (see Recipes)
4. Differentiation media 3 (Di-CLFR) (100 mL) (see Recipes)
5. Differentiation media 4 (Di-LSW) (100 mL) (see Recipes)
6. Differentiation media 5 (Di-HF) (100 mL) (see Recipes)
7. Differentiation media 6 (Di-H) (100 mL) (see Recipes)
8. FACS buffer (25 mL) (see Recipes)
9. Blocking buffer (20 mL) (see Recipes)
10. Papain solution (30 mL) (see Recipes)
11. Sodium citrate solution (0.1 M) (see Recipes)
12. Citric acid solution (0.1 M) (see Recipes)
13. EFTA stock solution 0.5 M pH 8.0

**Recipes**

1. **Coating solution (100 mL)**
  **Table:** 

Reagent	Final concentration	Quantity or Volume
EtOH	95%	95 mL
ddH_2_O	n/a	5 mL
Poly(methacrylacid-2-hydroxyethylester)	120 mg/mL	1.2 g
Total	n/a	100 mL
  The coating solution is not sterilized after mixing, as the high ethanol content makes contaminations unlikely. After application to the culture plate, it is sterilized by UV treatment. Alternatively, the coating solution can be sterilized through a 0.25 μm filter.
2. **Differentiation media 1 (Di-CL) (100 mL)**
  **Table:** 

Reagent	Final concentration	Quantity or Volume
DMEM/F12	n/a	96 mL
L-Glutamine	2 mM	1 mL
ITS-G	n/a	1 mL
NEAA	n/a	1 mL
P/S	500 U/mL	1 mL
CHIR-99021	3 μM	100 μL
LDN-193189	0.5 μM	100 μL
Total	n/a	100 mL
3. **Differentiation media 2 (Di-CLF) (100 mL)**
  **Table:** 

Reagent	Final concentration	Quantity or Volume
DMEM/F12	n/a	96 mL
L-Glutamine	2 mM	1 mL
ITS-G	n/a	1 mL
NEAA	n/a	1 mL
P/S	500 U/mL	1 mL
CHIR-99021	3 μM	100 μL
LDN-193189	0.5 μM	100 μL
bFGF	10 ng/mL	50 μL
Total (optional)	n/a	100 mL
4. **Differentiation media 3 (Di-CLFR) (100 mL)**
  **Table:** 

Reagent	Final concentration	Quantity or Volume
DMEM/F12	n/a	96 mL
L-Glutamine	2 mM	1 mL
ITS-G	n/a	1 mL
NEAA	n/a	1 mL
P/S	500 U/mL	1 mL
CHIR-99021	3 μM	100 μL
LDN-193189	0.5 μM	100 μL
bFGF	5 ng/mL	25 μL
Retinoic acid	10 nM	10 μL
Total	n/a	100 mL
5. **Differentiation media 4 (Di-LSW) (100 mL)**
  **Table:** 

Reagent	Final concentration	Quantity or Volume
DMEM/F12	n/a	96 mL
L-Glutamine	2 mM	1 mL
ITS-G	n/a	1 mL
NEAA	n/a	1 mL
P/S	500 U/mL	1 mL
LDN-193189	0.5 μM	100 μL
SHH	34 ng/mL	100 μL
WNT1A	20 ng/mL	200 μL
Total	n/a	100 mL
6. **Differentiation media 5 (Di-HF) (100 mL)**
  **Table:** 

Reagent	Final concentration	Quantity or Volume
DMEM/F12	n/a	96 mL
L-Glutamine	2 mM	1 mL
ITS-G	n/a	1 mL
NEAA	n/a	1 mL
P/S	500 U/mL	1 mL
HGF	10 ng/mL	100 μL
bFGF	10 ng/mL	50 μL
Total	n/a	100 mL
7. **Differentiation media 6 (Dix-H) (100 mL)**
  **Table:** 

Reagent	Final concentration	Quantity or Volume
DMEM/F12	n/a	96 mL
L-Glutamine	2 mM	1 mL
ITS-X	n/a	1 mL
NEAA	n/a	1 mL
P/S	500 U/mL	1 mL
HGF	10 ng/mL	100 μL
Total	n/a	100 mL
8. **FACS-Buffer (25 mL)**
  **Table:** 

Reagent	Final concentration	Quantity or Volume
PBS 1×	n/a	18.24 mL
EDTA	2 mM	0.1 mL
BSA	2%	6.66 mL
Total	n/a	25 mL
9. **Blocking buffer (20 mL)**
  **Table:** 

Reagent	Final concentration	Quantity or Volume
PBS 1×	n/a	18.24 mL
BSA	2%	5.33 mL
Goat serum (NGS)	10%	2 mL
Total	n/a	20 mL
10. **Papain solution (30 mL)**
  **Table:** 

Reagent	Final concentration	Quantity or Volume
DMEM/F12	n/a	30 mL
Papain	0.3 M	36 mg
EDTA	2%	8 mg
L-Cysteine	10%	8 mg
Total	n/a	30 mL
11. **Sodium citrate solution (0.1 M)**
  **Table:** 

Reagent	Final concentration	Quantity or Volume
Sodium citrate	0.1 M	29.41 g
ddH_2_O	n/a	1,000 mL
Total	n/a	1,000 mL
12. **Citric acid solution (0.1 M)**
  **Table:** 

Reagent	Final concentration	Quantity or Volume
Citric acid	0.1 M	21.1 g
ddH_2_O	n/a	500 mL
Total	n/a	500 mL

**Laboratory supplies**

1. Cell culture plate, 24 well, surface: standard, flat base (SARSTEDT, catalog number: 83.3922)
2. Cell strainers 40 μm (SARSTEDT, catalog number: 83.3945.040)
3. Parafilm (Bemis, catalog number: 11772644)
4. Serological pipette, with tip, plugged, 5 mL, sterile (SARSTEDT, catalog number: 86.1253.025)
5. Serological pipette, with tip, plugged, 10 mL, sterile (SARSTEDT, catalog number: 86.1254.025)
6. Tissue culture dish, 35 × 10 mm, surface: standard (SARSTEDT, catalog number: 83.3900)
7. Tissue culture dish, 100 × 20 mm, surface: standard (SARSTEDT, catalog number: 83.3902)

## Equipment

1. Cell culture microscope (Olympus, model: CKX41)
2. Leica modular stereo microscope (Leica, model: MZ10F)
3. CryoStar NX50 (Thermo Scientific, catalog number: 957210)
4. FACS sorter (Beckman Coulter, MoFlo Astrios EQ Cellsorter, catalog number: B52102)
5. Neubauer chamber (Marienfeld, catalog number: 0640131)
6. ZEISS slide scanner (Carl Zeiss Microscope, model: Axio Scan.Z1)
7. ZEISS confocal laser scanning microscope (Carl Zeiss Microscope, model: LSM 800)
8. Eppendorf centrifuge 5804R (Eppendorf, catalog number: EP022628146)

## Software and datasets

1. Summit (Beckman Coulter, version: 6.3.1)
2. Kaluza Analysis (Beckman Coulter, version: 2.1)
3. Zen Lite Blue (Carl Zeiss Microscope, version: 4.0.3, June 2021)
4. R v4.2 ( https://www.r-project.org/ , April 2022)

## Procedures

- **Human pluripotent stem cell maintenance**
  1. Human PSCs are cultured as colonies on 35-mm dishes, coated with 4% Matrigel with TeSR^TM^-E8^TM^ or StemFlex^TM ^medium. Cells are passaged at 70% confluency.
  2. Day -3: Split cells to 1:3/1:4 ratio of a 70% confluent hiPSC culture.
    - Discard media and rinse the dish with PBS to remove non-adherent cells and incubate them with TrypLE for chemical dissociation for 3–5 min at 37 °C.
    - Stop dissociation using basal media DMEM/F12.
    - Spin the cells at 400 rpm for 5 min, discard the DMEM/F12 resuspended, and plate the cells in TeSR^TM^-E8^TM^ supplemented with 10 µM Y-27632 onto new Matrigel-coated 35 mm dishes. (Dissolve 0.5 mL of Matrigel in 24.5 mL of DMEM/F12. Add 2 mL of the solution to a 35 mm dish and polymerize the Matrigel at 37 °C for 2 h or overnight at room temperature (RT), followed by UV sterilization before use.)
    - Culture the cells for two days and exchange TeSR^TM^-E8^TM^ daily. Use 2 mL of media for a 35 mm dish.
- **Generation of embryoid bodies (EBs) and differentiation of organoids**
  1. Day -4: Prepare low-attachment coated plates by adding 150 μL of coating solution into each 24-well plate well. Let the ethanol evaporate overnight and store plates at RT until use.
  2. Day -3/-2: Passage hiPSCs culture at 70%–80% confluency using the enzymatic dissociation approach to 1:3/1:4 ratio. The following day, refresh media without Rock inhibitor and, if:
    - Culture is at 30%–40% confluence, proceed with generating EBs the following day.
    - Culture is above 50% confluence, proceed with generating EBs the same day after refreshing the media without Rock inhibitor and culture the cells for at least 4–5 h before dissociating again for performing hanging drop method to generate EBs.
  3. Day -1: Generation of EBs via hanging drop method:
    - Dissolve 40 mg of PVA in 10 mL in TeSR^TM^-E8^TM^/StemFlex^TM^, followed by sterilization via filtration (0.25 μM) and supplemented with 10 μM Y-27632 and 1% P/S.
    - Discard media, rinse the dish with PBS to remove non-adherent cells, and incubate them with TrypLE for chemical dissociation for 3–5 min at 37 °C.
    - Stop dissociation using basal media DMEM/F12.
    - Spin the cells at 400 rpm for 5 min, discard the DMEM/F12, and resuspend in TeSR^TM^-E8^TM^/StemFlex^TM^ supplemented with 10 μM Y-27632 and 1% P/S and PVA.
    - Count cells and adjust cell density to 200,000 cells/mL (=4,000 cells/20 μL).
    - Use the lid of a 10 cm cell culture dish and place 20 μL drops onto it. Fill the dish with PBS for humidified condition and place the lid again on the dish.
    - For EB formation, incubate the 10 cm cell culture dish overnight in the incubator at 37 °C and 5% CO_2_.
  4. Day 0:
    - EBs should have a size of 200–250 μm.
*Note: If a cloud of dead single cells surrounds the EB, and the EB is below 200 μm in size, while repeating step B3, increase the concentration of Rock inhibitor to 1.5–3× to enhance cell survival.*
    - Thaw Matrigel on ice in the fridge; it should stay cold (<4 °C) to avoid premature polymerization throughout the process.
    - Wash EBs into a dish using DMEM/F12.
*Note: The following step takes place under a stereoscope outside the hood after thorough disinfection of the used area. The application of 1.5× P/S in the medium prevents possible contaminations.*
    - Place individual EBs onto a Parafilm^®^ embedding surface using a 200 μL pipette. Remove excessive media but never leave EB without media (should remain approximately 5 μL of DMEM/F12). Well-formed EBs should not take any harm from it.
    - Place a 30 μL Matrigel drop onto each EB, resuspend to ensure a homogeneous mix of EBs within the Matrigel droplet, and place the EB in the center of the drop using a 200 μL pipette.
*Note: To ensure homogeneous Matrigel polymerization: Resuspend 2–3× up and down and only place the EB within the Matrigel. Matrigel polymerizes faster due to the light source of the stereoscope.*
    - Incubate the embedded EBs in the incubator at 37 °C for 20–25 min.
*Note: The following step takes place under a cell culture hood.*
    - Wash EBs into a 10 cm cell culture dish using DMEM/F12.
*Note: Embedded EB droplets appear reddish due to the Matrigel. Use fresh DMEM/F12 for better visualization when collecting them with a cut-off 1,000 μL pipette tip. The pipette tips are not coated before handling cells, EBs, or SMOs throughout the protocol.*
    - Prepare Di-CL media and place 1 mL in each low-attachment coated-plate well and warm it in the incubator.
    - Use one cut-off 1,000 μL pipette tip to transfer embedded EBs into a low-attachment coated-plate well and incubate them at 37 °C and 5% CO_2_.
    - Exchange media partly every day.
*Note: Developing organoids are quite small. Control after media change if organoids are still present. To avoid destroying organoids during media change, hold the culture plate at 45 degrees and carefully remove media by pointing at the top of the well and aspirating slowly. Organoids should never be dried out during media change.*
*Important: Because myogenesis and migration take place within the Matrigel droplet, damaging the Matrigel during media change should be avoided at all times.*
*Note: This step requires extra caution to not lose or destroy the developing organoids, which can be challenging for beginners in all organoid protocols. The poor visibility of the embedded EB is a considerable criterion. A dark base under the cell culture plate helps to make it easier to recognize. Tilting the plate by just under 45° to the experimenter causes the organoids to shift to the lower edge and thus reduces the probability of damaging them.*
  5. Day 3: Change media from Di-CL to Di-CLF media by removing 75% and adding the same volume of media.
  6. Day 5: Change media from Di-CLF to Di-CLFR media by removing 75% and adding the same volume of media.
  7. Day 7: Change media from Di-CLFR to Di-LSW media by removing 75% and adding the same volume of media. Change media every second day.
  8. Day 11: Change media from Di-LSW to Di-HF media by removing 75% and adding the same volume of media. Change media every second day.
  9. Day 15: Change media from Di-HF to DiX-H media by removing 75% and adding the same volume of media. Change media every second day.
  10. From Day 30 on: Change media every third day.
    Steps 5–10 are depicted in the protocol outline of Figures 1A and 3A.
    **Figure 1.**
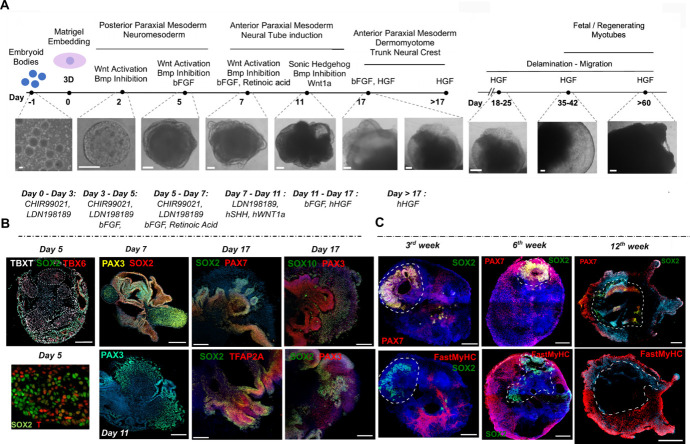
Representative images of different stages of skeletal muscle organoid development. A. Brightfield images of myogenic development stages, with corresponding cytokines/growth factor applications. B. Representative immunocytochemistry pictures of mesodermal, neural, paraxial mesodermal, and neural crest origin during early stages. C. Representative immunocytochemistry pictures depict neural lineage arrest, myogenic progenitor migration, and skeletal muscle organoid formation at more mature stages following organoid culture progression. Dashed lines indicate the initial EB embedding site and growth before migration takes place. Scale bars: 500 μm in (C), 200 μm in A (Day 18–Day 60) and B (Day 17), 100 μm in A (Day -1–Day 17) and B (Day 5, Day 7, Day 11) (modified from Mavrommatis et al. [18]).
- **Immunostaining of organoids at different stages**
  1. Fix organoids from different stages in 4% PFA (in PBS) overnight at 4 °C under shaking conditions.
  2. Following PFA fixation, dehydrate organoids with 30% sucrose solution in PBS overnight and embed them in Tissue-Tek^®^ O.C.T. compound.
  3. Acquire cryosections of 20–30 μm thickness on a cryotome.
  4. For the immunostaining process, rehydrate cryosections with PBS, permeabilize them first with 0.1% Tween-20 (10 min) in PBS, and then rinse 3× with 1× PBS.
  5. Permeabilize further the cryosections with 0.1% Triton X-100 in PBS (10 min) and rinse 3× with PBS.
  6. Block cryosections with the blocking buffer for 1 h at RT.
*Note: For primary antibodies of goat origin, use blocking solution that contains only 2% BSA.*
  7. Incubate with primary antibodies overnight at 4 °C and secondary antibodies for 2 h at RT.
  8. Mount cryosections with mounted media containing DAPI.
  9. Acquire images on a confocal microscope and analyze them using Zen Lite Blue. Cryosections can be stable for months in the fridge; however, this cannot be generalized for all antibodies. Representative images are shown in Figures 1B and 1C.
- **Profiling skeletal muscle organoids from mature stages with single-cell RNA sequencing**
  1. Prepare single cells by incubating for 1 h with papain solution. Alternatively, an incubation with TrypLE Select for 15 min at 37 °C followed by mechanical dissociation by pipetting up and down using a 1000 μL pipette upon resuspension also generates single cells suitable for scRNAseq pipeline.
*Note: For TrypLE gentle dissociation, cut the 1 mL pipette tip before resuspending.*
  2. Upon dissociation, estimate cell number and viability. Resuspend cells in a solution containing 0.5% BSA in PBS to reach a concentration of 390 cells per μL.
  3. Prepare cDNA libraries using the Chromium Single Cell 3′ Reagent kit (v3) (Single Cell 3′ Library & Gel Bead Kit v3, Single Cell B Chip Kit, and i7 Multiplex Kit) or an equivalent method according to the manufacturer’s instructions.
  4. Sequence cDNA libraries on an Illumina HiSeq 3000 or equivalent method with 150 bp paired-end reads.
  5. Exemplary downsteam bioinformatic analysis in R is illustrated in Figure 2.
    **Figure 2.**
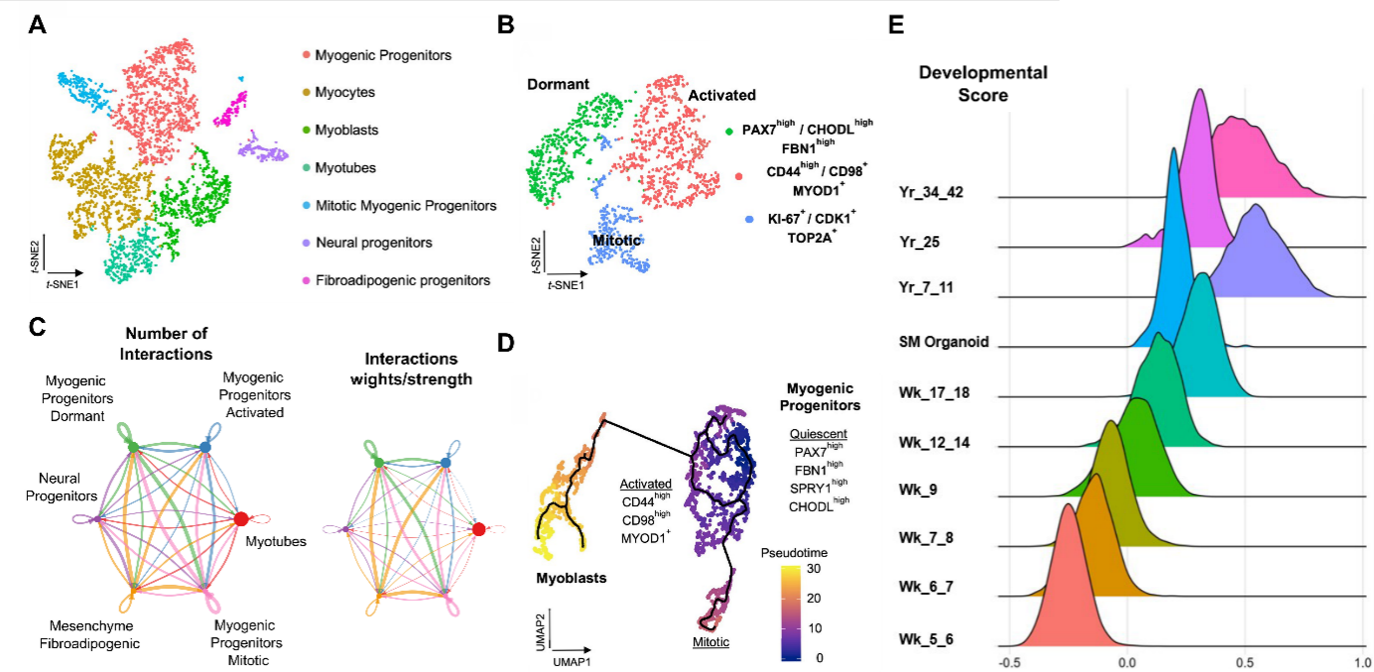
Representative single-cell RNA-seq profiling of late fetal myogenic progenitors at mature stages during skeletal muscle organoid development. A. *t*-SNE visualization of color-coded clustering (n = 4323 cells) at 12 weeks post differentiation highlights the predominant presence of skeletal muscle lineage, represented by clusters corresponding to myogenic progenitors (n = 1625 cells, 37% of total population) in non-dividing (n = 1317 cells) and mitotic (n = 308 cells) state, myoblasts (n = 731 cells), myocytes (n = 1147 cells), and myotubes (n = 442). Additionally, mesenchymal and neural lineages are represented by two smaller clusters of fibroadipogenic (n = 165 cells) and neural (n = 213 cells) progenitors, respectively. B. *t*-SNE plot visualization of color-coded clustering indicates myogenic progenitor subcluster with distinct molecular signatures: “dormant” PAX7^high^/CHODL^high^/FBN1^high^, “activated” CD44^high^/CD98^+^/MYOD1^+^, and “mitotic” KI-67^+^/CDK1+/TOP2A. C. Circle plot illustrates the aggregated cell–cell communication network for all clusters at week 12 of human skeletal muscle organoids development. Circle sizes are proportional to the number of cells in each cell group and edge width represents the communication probability. D. Pseudo-time ordering for myogenic progenitors and myoblast-corresponding clusters highlight distinct developmental trajectories promoting myogenic commitment and self-renewal. E. Ridge plots of developmental score distribution of myogenic progenitors across in vivo or in vitro stages, based on the difference between upregulated satellite cell and embryonic markers from human reference atlases for week (Wk)-5–18 embryonic and fetal stages, years (Yr)-7–42 adult satellite cells and skeletal muscle organoids (modified from Mavrommatis et al. [18]).
- **In vivo potential of SMO-derived skeletal muscle progenitors**
  1. FACS isolation of CD82-positive skeletal muscle progenitor cells.
    - Dissociate the SMOs with TrypLE Select for 45 min at 37 °C including a mechanical dissociation step after 25 and 45 min by pipetting up and down using a 1,000 μL pipette. The bulges of SMOs on day 84 are enriched with progenitors (Figure 3B, day 84).
    - Pipetting up and down 3–4 times every 15–20 min helps to dissociate the SMOs into a single-cell suspension.
    - Stop the reaction with FACS buffer by adding 3–4 times the volume of TrypLE Select.
    - Discard cell clumps by filtration through a 40 μm cell strainer followed by a centrifugation step.
    - Label the cells with PE anti-human CD82 antibody and APC anti-human CD56 (NCAM) antibody in FACS buffer (5 μL of each antibody in 100 μL of FACS buffer for 1 × 10^6^ cells) and incubate the cell suspension on ice for 1 h, followed by a centrifugation step at 400 rpm for 5 min at RT.
    - Resuspend cells in FACS buffer supplemented with DAPI.
    - Use unstained SMO cells as baseline controls to exclude autofluorescence and sort the stained cells using a FACS sorter (Figure 3C).
    - FACS data are captured using summit software and analyzed using Kaluza Analysis software.
  2. Transplantation of CD82-positive cells.
    - Cause an injury in the tibialis anterior (TA) muscle of 2–3-months-old male HsdCpb:NMRI-Foxn1nu mice by injecting 10 μL of CTX (40 ng/mL) 24 h before transplantation.
    - Under anesthesia, inject 1 × 10^5^ cells into the TA muscle on one side of the mice.
*Note: The injection was applied methodically as pictured in Feige & Rudnicki [27], Figure 4.*
    - After six weeks, sacrifice the mice and isolate the TA.
    - Fix the TA in 4% PFA (in PBS) followed by embedding into O.C.T. compound media.
    - Slice the embedded TA in cross sections of 10–20 μm thickness using a CryoStar NX50.
    - Rehydrate slices with PBS, heat in citrate buffer (final concentration 19 nM, mix 82 mL of sodium citrate solution and 18 mL of citric acid solution plus 900 mL of ddH_2_O) until the buffer boils, and cook continuously for 15 min at 95 °C for antigen heat retrieval.
    - Treat the slices with an AffiniPure Fab Fragment Goat Anti-Mouse IgG for 60 min at RT to prevent unspecific bindings.
    - Permeabilize the slice with 1% (vol/vol) Triton X-100 and 125 mM glycine in PBS for 20 min at RT.
    - After blocking with blocking buffer for 60 min at RT, incubate the slides with the primary antibody overnight at 4 °C.
    - After washing three times for 10 min with PBS, incubate the slides with the secondary antibody for 2 h at RT.
    - Use Zeiss Scan.Z1 to scan the slides and process them using Zen Lite Blue (Figure 3D).
  **Figure 3.**
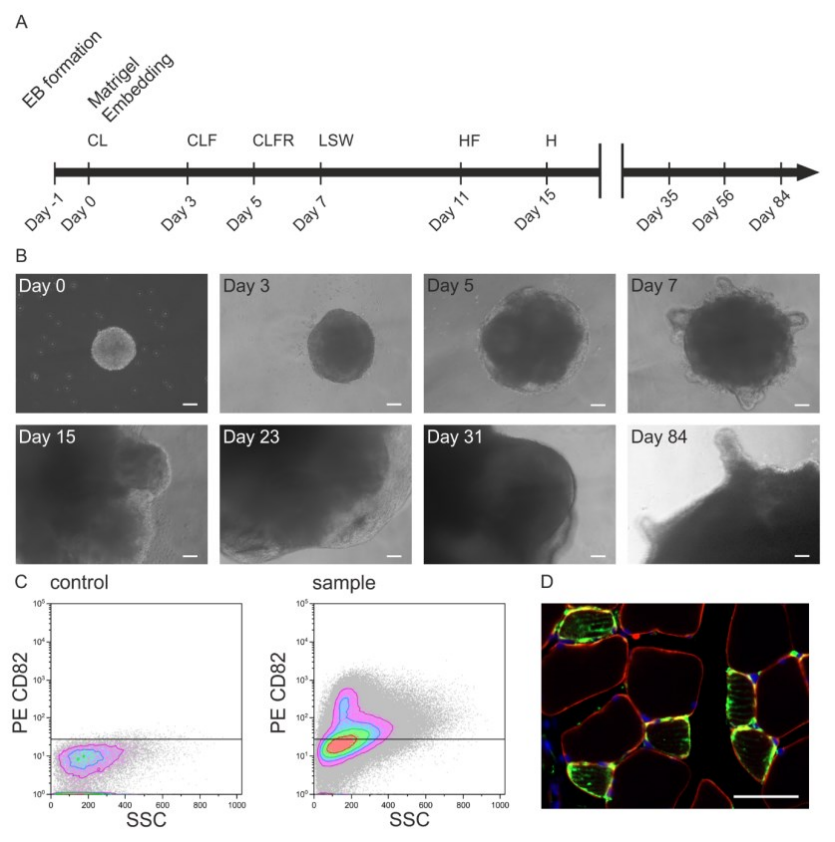
In vivo potential of skeletal muscle organoids (SMO)-derived skeletal muscle progenitors. A. Illustration of the differentiation protocol including the timeline of culture media addition and factor incubation (C: CHIR99021, L: LDN193189, F: bFGF, H: HGF, R: retinoic acid, S: Sonic hedgehog, W: WNT1A). B. Brightfield microscopy images of iPSC-derived SMOs on the selected days of a 84-day culture period (scale bar: 100 μm). C. FACS of organoid-derived CD82-positive skeletal muscle progenitors and D. their evaluation 6 weeks after transplantation into the CTX-injured tibialis anterior of an immunodeficient mouse [staining with huLamin A/C (green), dystrophin (red), DAPI (blue); scale bar 50 μm].

## Validation of protocol

The reproducibility of the protocol has been described within various paragraphs of the corresponding article Mavrommatis et al. (2023). The organoid approach was evaluated with six hiPSC lines with independent genetic backgrounds, with more than five independent derivations per line, for the control line (CB CD34+) with more than 20 derivations, always obtaining similar results. The organoids show very reproducible sizes during their development (Figure 1B of Mavrommatis et al. [18]). To further evaluate the reproducibility of organoid development, diffusion map analysis on qPCR-based expression analysis of 32 genes was applied at early stages, as well as integrative analysis on scRNAseq datasets of mature stages of organoid development from four independent iPSC lines. The data indicate highly conserved cluster representation of myogenic progenitors at all states, together with skeletal muscle myofibers, fibroadipogenic progenitors, and neural progenitors-related clusters (Figure 4, Supplemental Figure 6, Mavrommatis et al. [18]).

## Data analysis

RNA sequencing datasets produced by Mavrommatis et al. 2020, 2023 [17,18] are deposited in the Gene Expression Omnibus (GEO) under accession code GSE147514. To review GEO accession GSE147514: Go to https://www.ncbi.nlm.nih.gov/geo/query/acc.cgi?acc=GSE147514.
